# Resistance Training Diminishes the Expression of Exosome CD63 Protein without Modification of Plasma miR-146a-5p and cfDNA in the Elderly

**DOI:** 10.3390/nu13020665

**Published:** 2021-02-19

**Authors:** Brisamar Estébanez, Nishant P. Visavadiya, José A. de Paz, Michael Whitehurst, María J. Cuevas, Javier González-Gallego, Chun-Jung Huang

**Affiliations:** 1 Institute of Biomedicine (IBIOMED), University of León, 24007 León, Spain; japazf@unileon.es (J.A.d.P.); mj.cuevas@unileon.es (M.J.C.); jgonga@unileon.es (J.G.-G.); 2 Exercise Biochemistry Laboratory, Department of Exercise Science and Health Promotion, Florida Atlantic University, Boca Raton, FL 33431, USA; nvisavadiya@fau.edu (N.P.V.); whitehur@fau.edu (M.W.)

**Keywords:** aging, cell-free DNA, exercise-promoted exosomes, extracellular vesicles, inflammaging, microRNAs, physical activity, strength training

## Abstract

Aging-associated inflammation is characterized by senescent cell-mediated secretion of high levels of inflammatory mediators, such as microRNA (miR)-146a. Moreover, a rise of circulating cell-free DNA (cfDNA) is also related to systemic inflammation and frailty in the elderly. Exosome-mediated cell-to-cell communication is fundamental in cellular senescence and aging. The plasma changes in exercise-promoted miR-146a-5p, cfDNA, and exosome release could be the key to facilitate intercellular communication and systemic adaptations to exercise in aging. Thirty-eight elderly subjects (28 trained and 10 controls) volunteered in an 8-week resistance training protocol. The levels of plasma miR-146a-5p, cfDNA, and exosome markers (CD9, CD14, CD63, CD81, Flotillin [Flot]-1, and VDAC1) were measured prior to and following training. Results showed no changes in plasma miR-146a-5p and cfDNA levels with training. The levels of exosome markers (Flot-1, CD9, and CD81) as well as exosome-carried proteins (CD14 and VDAC1) remained unchanged, whereas an attenuated CD63 response was found in the trained group compared to the controls. These findings might partially support the anti-inflammatory effect of resistance training in the elderly as evidenced by the diminishment of exosome CD63 protein expression, without modification of plasma miR-146a-5p and cfDNA.

## 1. Introduction

The aging process is characterized by the accumulation of failures at the molecular and cellular level, which leads to functional decline of tissues and organs [1] and deterioration of physiological functions [2]. The age-related changes in the immune system, also known as immunosenescence, result in a low-grade pro-inflammatory condition referred as inflammaging. This inflammaging is mainly attributed to a somatic cellular senescence-associated secretory phenotype (SASP) [3], in which senescent cells secrete high levels of inflammatory mediators, such as cytokines and microRNAs (miRNAs or miRs) [4,5]. Specifically, mitochondrial miR-146a-5p is a well-established senescence-associated inflammatory mediator for human umbilical vein endothelial cells (HUVECs) [6]. Furthermore, miR-146a-5p has also been identified as an important regulator of DNA damage response (DDR)—senescence—inflammation crosstalk [7]. In this regard, cell-free DNA (cfDNA) has been recognized as a damage-associated molecular pattern (DAMP), inducing NF-κB inflammatory response [8], and elevated levels of circulating cfDNA have been found to reflect systemic inflammation and frailty in the elderly [9,10]. Although cfDNA is released into circulation through various mechanisms under a wide range of biological and environmental factors [11], the accumulation of cfDNA depends on its release from cells, free or in extracellular vesicles (EVs), its binding in protein aggregates, and its uptake by surrounding cells [12,13,14]. EVs are critical mediators in intercellular communication and play important roles in cellular senescence and aging [4]. Eitan and colleagues [15] have discovered a lower level of plasma EVs in the elderly compared to young individuals, possibly due to increased internalization of peripheral blood mononuclear cells (PBMCs).

Emerging evidence suggests that exercise is an effective intervention to delay the progression of aging-related diseases [16,17,18]. The release of exercise-promoted exosomes could be the key to facilitating intercellular communication [19] and systemic adaptations to exercise [20] in aging [21] and other conditions and diseases, such as type 2 diabetes [22,23], cardiovascular diseases [24,25,26], and sarcopenia [27]. Thus far, the literature has demonstrated the release of exosome markers (Alix, CD63, CD81, and Flotillin [Flot]-1) regardless of exercise protocols [21,24,25,26,28,29,30,31,32,33,34], although the effect of exercise training on these exosome cargo remains to be elucidated in the elderly. Additionally, several studies have investigated the effects of exercise on the release of age-related biomarkers, such as cfDNA, or inflammaging-related mitochondrial miRNAs, such as miR-146a-5p. For instance, the plasma level of cfDNA was decreased in older adults following a combination of 6-month resistance training and nutritional supplementation (protein and micronutrients [e.g., vitamins C and E]) [35]. Furthermore, Morais Junior et al. [36] examined acute effect of 1-week resistance exercise protocol in elderly subjects and found an increase in the circulating level of miR-146a-5p. However, there is currently limited literature examining a long-term resistance training program on the expression of inflammaging-related miR-146a in plasma from elderly people.

Taking into consideration all aforementioned observations, this study aimed to examine the effects of both aging and an 8-week resistance training on the levels of plasma miR-146a-5p, cfDNA, and exosome cargo (CD9, CD14, CD63, CD81, Flot-1, and VDAC1) in elderly subjects. The potential relationships of miR-146a-5p and cfDNA with these exosome markers were also investigated.

## 2. Materials and Methods

### 2.1. Subjects Characteristics

Fifty healthy elderly (*N* = 38; 16 males and 22 females) and young (*N* = 12 males) subjects volunteered to participate in the study. The exclusion criteria included the subjects aged under 70 and over 85 with any known inflammatory diseases/conditions (e.g., dyslipidemia, hypertension, and diabetes mellitus) or the use of any medication known to affect the inflammatory status in the past 6 months prior to or during the study. Moreover, none of the female participants were taking any hormonal treatment, either before or at the time of the study. Participants did not have any experience in resistance training, and they were asked to maintain their physical activity routines during the study period. Prior to participation in this study, all subjects completed the informed consent form, and a medical screening, including anthropometric analysis, the physical activity readiness questionnaire (PAR-Q), a risk factor quiz, blood pressure measurements, and a basal electrocardiogram test, was performed in all the elderly participants. Then, the elderly subjects were randomly assigned to either a training group (TG; *N* = 28; 15 males and 13 females) or a control group (CG; *N* = 10; 1 male and 9 females). The participants from the TG followed an 8-week resistance training, whereas the control group maintained their normal daily routines throughout the experiment. Finally, this study followed the principles of the Declaration of Helsinki, and the local ethics committee approved all procedures.

### 2.2. Maximal Strength Assessment

The one-repetition maximum (1RM) test was performed on a BH^®^ Fitness Nevada Pro-T machine (Madrid, Spain), with an angle of 100° between the plane of the seat and the back. Before all testing procedures, the subjects participated in a general warm-up period that consisted of 10 repetitions with 40% of their body weight. If this weight was lifted with the proper form, each participant performed a new trial in which the load was increased by approximately 5 to 20 kg, and the subject attempted two repetitions with the selected weight. If successful, the testing continued until a 1RM lift was determined. The 1RM was determined across three to six sets, excluding warm-up. All increases in weight were dependent upon the rating of perceived exertion in each attempt, which was assessed using the rating of perceived exertion (RPE) OMNI-resistance exercise scale (OMNI-RES) (0–10) [37], and rest periods between each attempt were set around 3 min between attempts. For the 1RM test of leg extension, the participants were seated with a knee flexion of 90°. Before the test, the lever arm of the knee extension machine was aligned with the center of rotation of the knee joint. The tibial pad was individually adjusted proximally to the medial malleolus on the lower extremity for every subject. The range of motion of the knee joint began at 90° and ended around 180°. After 10 min of rest, the 1RM seated bench press test was performed with the same protocol and material as described above. In this exercise, the participants were seated with arms in abduction at 90° and with elbows bent at 90°. The range of motion of the elbow joint began at 90° and ended around 180°.

### 2.3. Resistance Exercise Training

Subjects from the TG completed 16 resistance training sessions over 8 weeks (2 sessions per week), with a minimum of 48 h between sessions. After a 10 min warm-up on a cycle ergometer, the participants performed eight different resistance exercises, including leg press, ankle extension, bench press, leg extension, biceps curl, pec deck, high pulley traction, and dumbbell lateral lift in the same exercise devices. The participants performed three sets of 12-8-12 repetitions for each exercise. The load of the first four exercises started at 40% 1RM, increasing load by 5% each week. For the last four exercises, the load was prescribed according to the RPE with the OMNI-RES from 1 (light) to 10 (intense) as follows: the first week with an intensity of scale 5, the next three weeks with the intensity increased from scale 5 to 6, and followed by an increase to scale 8 in the last four weeks [38]. Between each repetition, the participants had 2–3 min of rest, while a 3 min rest was given between each exercise.

### 2.4. Blood Sampling

Venous blood samples (30 mL) were obtained from the antecubital vein of elderly subjects using the ethylenediamine tetraacetic acid (EDTA) anticoagulant Vacutainer™ system (BD, Franklin Lakes, NJ, USA) approximately 5–6 days prior to and after the completion of training period. In young subjects, the blood samples were only collected prior to the training intervention. To avoid circadian effects, all samples were collected between 08:00 a.m. and 09:00 a.m. following an overnight fast. The participants were required to avoid any intense exercise prior to each blood collection. No caffeine or alcohol was allowed during the experiment. All blood samples were centrifuged at 300× *g* for 10 min at 4 °C, and plasma was stored at −80 °C until further analysis.

### 2.5. miRNA Extraction, Reverse Transcription, and RT-PCR

Total miRNA was extracted from 200 μL of plasma with QIAGEN miRNeasy Serum/Plasma Kit (QIAGEN, Hilden, Germany) following the manufacturer’s protocol. Normalization of sample-to-sample variation in miRNA quantification was achieved by spiking in a mixture of 5 μL of 5 fmol/μL *Caenorhabditis elegans* miRNA-39 (cel-miR-39) and 0.7 μL of RNA, MS2 (Roche, Indianapolis, IN, USA) to each plasma sample during extraction. A 5 μL of extracted miRNA was then reverse-transcribed with the TaqMan MicroRNA Reverse Transcription Kit (ABI, Foster City, CA, USA) and each respective primer (miR-146a-5p, assay ID: 000468; miR-16, assay ID: 000391; cel-miR-39, assay ID: 000200; ABI, Foster City, CA, USA) according to the manufacturer’s protocol. Subsequently, RT-PCR was used to quantify miR-146a and internal positive control (miR-16), along with the spike-in control (cel-miR-39) with TaqMan MicroRNA assays and their corresponding primer probes (ABI, Foster City, CA, USA) following the manufacturer’s protocol. For each RT-PCR plate, target samples were analyzed in duplicates. All reverse transcription and RT-PCR were performed on the CFX Connect Real-Time PCR Detection System (Bio-Rad, Hercules, CA, USA). Finally, the miR-146a expression were normalized by subtracting the average of miR-16 and cel-miR-39 values.

### 2.6. cfDNA Assay

Plasma cfDNA was analyzed by the fluorescent SYBR™ Gold nucleic acid gel stain (Cat# S11494, Thermo Fisher Scientific, Waltham, MA, USA) method as described previously [39]. Briefly, an 80 µL of diluted fluorochrome stain (diluted first at 1:1000 in DMSO and followed by a 1:8 dilution in PBS) was added to the 20 µL of plasma sample or DNA standard solution in black 96-well plate (Cat# 655906, Greiner Bio-One, Frickenhausen, Germany). Fluorescent formation was recorded at 485 nm (excitation) and 535 nm (emission) by Synergy™ HTX Multi-Mode spectrofluorometer (BioTek Instruments, Winooski, VT, USA). The standard curve was established (0 to 5000 ng/mL) through serial dilution of UltraPure™ salmon sperm DNA (Cat# 15632011, Thermo Fisher Scientific, Waltham, MA, USA).

### 2.7. Exosome Isolation

The exosome isolation was performed by modifying the protocol of Théry et al. [40] (protocol suggested by Miltenyi Biotec: DS MACSPlex Exosome Kit human 130-108-813). Briefly, a 1.3 mL plasma sample was diluted in an equivalent volume of phosphate buffered saline (PBS) and centrifuged at 2000× *g* for 30 min at room temperature. Subsequently, the supernatant was centrifuged at 10,000× *g* for 45 min at 4 °C (Sorvall RC-5B Superspeed Centrifuge, Du Pont Instruments, USA), and the new supernatant was ultracentrifuged at 100,000× *g* for 120 min at 4 °C (Optima XL-100K Ultracentrifuge, Beckman Coulter, Brea, CA, USA). Then, the precipitate was resuspended in a volume of PBS equivalent to the initial volume of plasma (1.3 mL) and filtered through 0.2 μm filters (Titan3™ PES (polyethersulfone) Syringe Filters, Thermo Fisher Scientific, Waltham, MA, USA). Subsequently, the filtering was subjected to a new ultracentrifugation at 100,000 × *g* at 4 °C (Optima XL-100K Ultracentrifuge, Beckman Coulter, Brea, CA, USA). The exosome precipitate was resuspended in 50 μL of 5% sodium dodecyl sulfate (SDS)-PBS with antiproteases (cOmplete™ Mini EDTA-free Protease Inhibitor Cocktail, Roche, Switzerland) and stored at −80 °C for further analysis.

### 2.8. Western Blot Analysis

DC™ protein assay (Bio-Rad, Hercules, CA, USA) was used to measure protein content of exosome samples in the microplate reader Synergy HT (BioTek, Winooski, VT, USA). Samples with 40 μg of protein were fractionated by SDS-PAGE on 12% polyacrylamide gels. Then, separated proteins were transferred to polyvinylidene difluoride (PVDF) membranes and pre-incubated in 5% non-fat milk for 60 min at room temperature to block non-specific binding. Afterwards, membranes were incubated overnight at 4 °C with specific primary antibodies. Antibodies against Flot-1 (Cat# sc-74566, RRID: AB_2106563), CD63 (Cat# sc-5275, RRID: AB_627877), CD81 (Cat# sc-166029, RRID:AB_2275892), CD9 (Cat# sc-13118, RRID:AB_627213), CD14 (Cat# sc-9150, RRID:AB_2074171), and VDAC1 (Cat# sc-8828, RRID:AB_793935) were purchased from Santa Cruz Biotechnology, CA, USA. Bound primary antibody was detected using a horseradish peroxidase (HRP)-conjugated secondary antibody (Dako, Glostrup, Denmark) along with an ECL-HRP kit (Luminol Reagent, Santa Cruz Biotechnology, CA, USA), and blots were exposed to autoradiography films and developed. Finally, the optical density (O.D.) of the specific bands was quantified with an imaging densitometer (Image J, Bethesda, MD, USA), and densitometric analyses were normalized to Ponceau S staining, 0.1 % (*w*/*v*) in 5% acetic acid (Sigma-Aldrich, St. Louis, MO, USA).

### 2.9. Statistical Analysis

All statistical analyses were performed using SPSS version 24 (SPSS Inc., Chicago, IL, USA). An independent test was used to examine the differences in subject characteristics and strength measurements between elderly trained vs. control groups. A Shapiro-Wilk test was used to confirm the normality of data for all outcome variables. As data were skewed, Mann–Whitney non-parametric analysis was used to compare the baseline levels and percent change (pre- vs. post-training) for the effects of aging (young vs. elderly) and resistance training (control vs. trained). Additionally, Tau-b de Kendall correlations were used to examine the relationships of plasma miR-146a-5p and cfDNA with exosome proteins. Differences were considered significant when *p* < 0.05. Data are presented as mean ± standard error of means (SEMs).

## 3. Results

### 3.1. Anthropometric and Strength Measurements of the Study Participants

The baseline anthropometric characteristics of the young and elderly groups (CG and TG) are reported in Table 1.

At baseline, our analyses did not show any differences in both 1RM bench press seated (54.222 ± 4.639 vs. 55.963 ± 4.181 kg; *p* = 0.824) and leg extension (54.700 ± 5.608 vs. 70.893 ± 4.607 kg; *p* = 0.063) strength assessments between CG and TG. However, following an 8-week resistance training protocol, a significant improvement in the percent change of 1RM bench press seated and leg extension strength was observed (Table 2).

### 3.2. Plasma Levels of miR-146, cfDNA, Total Exosome Protein Content, and Exosome Cargo at Baseline

**Figure 1:**
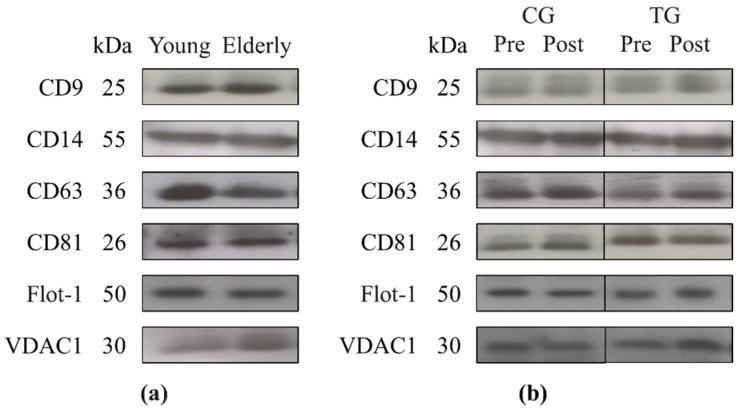
Representative Western blots showing the effects of aging (12 young vs. 38 elderly subjects) (**a**) and resistance training (CG (*N* = 10) vs. TG (*N* = 28)) (**b**) on Flot-1, CD63, CD81, CD9, CD14, and VDAC1 protein expression in plasma exosomes.

As shown in Table 3, no difference was found in the baseline levels of miR-146a-5p (*p* = 0.618) and cfDNA (*p* = 0.114) between young and elderly subjects measured by RT-PCR and fluorescent staining, respectively. Moreover, our analyses did not demonstrate any significant effects of aging (young vs. elderly groups; *p* = 0.140) in the total protein concentration of exosomes at baseline analyzed by the DC assay. However, Western blot showed that elderly subjects exhibited a lower expression of CD63 protein (*p* = 0.014) compared to young subjects at baseline (Figure 1a). Furthermore, our results showed comparable levels of other exosome markers (Flot-1 (*p* = 0.125), CD81 (*p* = 0.856), and CD9 (*p* = 0.262)) between young and elderly subjects. Similarly, no difference was found in the baseline levels of exosome-carried proteins CD14 (*p* = 0.486) and VDAC1 (*p* = 0.078) between both groups.

Additionally, both the CG and TG groups did not demonstrate any differences in the basal levels of plasma miR-146a-5p (*p* = 0.416) and cfDNA (*p* = 0.376) (Table 4). A comparable baseline level of total exosome protein concentration was also shown between both groups (*p* = 0.317). Finally, no differences were found in the baseline levels of plasma exosome markers: Flot-1 (*p* = 0.608), CD63 (*p* = 0.584), CD81 (*p* = 0.507), CD9 (*p* = 0.777) and exosome-carried proteins: CD14 and VDAC1 (*p* = 0.728 and *p* = 0.151, respectively) (Figure 1b).

### 3.3. Plasma Levels of miR-146a and cfDNA and Exosome Markers Following an 8-Week Resistance Training

As presented in Table 5, following an 8-week resistance training our results showed no differences in miR-146a-5p (*p* = 0.360) and cfDNA (*p* = 0.396) plasma levels. Likewise, there was no significant difference in the percent change of total exosome protein concentration between TG and CG (*p* = 0.947) with training. However, a significant difference in the percent change of CD63 protein was found with a diminished expression in TG vs. CG (*p* = 0.027) (Table 5/Figure 1b), although other exosome markers—Flot-1 (*p* = 0.784), CD81 (*p* = 0.842), and CD9 (*p* = 0.571)—did not show any changes. Additionally, the expression of exosome-carried proteins CD14 (*p* = 0.192) and VDAC1 (*p* = 0.433) also remained unmodified with resistance training. Finally, neither miR-146a-5p nor cfDNA was correlated with any exosome protein markers at baseline or in response to resistance training.

## 4. Discussion

This study aimed to investigate the effects of an 8-week resistance training on the release of plasma miR-146a-5p and cfDNA, along with the expression of exosome proteins, in the elderly. Our results demonstrated that the levels of miR-146a-5p and cfDNA remained unchanged with training. Furthermore, no changes in exosome markers (Flot-1, CD9, and CD81) and exosome-carried proteins (CD14 and VDAC1) were observed, whereas an attenuated CD63 response was found in the trained group compared to the controls. These findings might partially support the anti-inflammatory effect of resistance training in the elderly as evidenced by the diminishment of CD63 protein expression.

Upregulation of pro-inflammatory miRNAs can act to mitigate the excessive aging-associated pro-inflammatory response through a negative feedback [5]. As such, miR-146a-5p has been proved to inhibit the secretion of SASP factors, such as interleukin (IL)-6 and IL-8, via the downregulation of the IL-1 receptor-associated kinase 1 (IRAK1)-mediated inflammatory signaling pathway in primary human fibroblasts [41]. Unlike the studies demonstrating an upregulation of miR-146a-5p in senescent HUVECs [6] and fibroblasts [41], our results did not show any difference in the baseline level of plasma miR-146a-5p between young and elderly subjects. Elderly individuals with the severity of coronary stenosis have elevated plasma miR-146a-5p levels [42]. Furthermore, Guo et al. [43] reported a positive correlation between plasma miR-146a-5p and both carotid intima-media thickness and brachial-ankle pulse wave velocity in middle-aged to elderly diabetic patients. This discrepancy with our findings is most likely due to health status because the current study recruited only elderly individuals without a history of inflammatory and/or metabolic diseases, which may have eliminated some confounding factors compared with young adults. In addition, no change in the level of plasma miR-146a-5p was observed following 8 weeks of resistance training in this study. While there is limited literature regarding training-mediated miR-146a-5p response in the elderly, a decrease in the circulating level was found in middle-aged obese patients following a 3-month combined aerobic and resistance training [44]. However, acute intense exercise [45] showed an elevated miR-146a-5p expression in skeletal muscle 4 h into recovery, possibly mediating exercise-induced muscle inflammation/damage [46]. Thus, the differential expression in the level of miR-146a-5p to acute and chronic exercise may support the importance of physiological adaptations to exercise in the modulation of related inflammatory conditions.

cfDNA can act as a DAMP [47], triggering the immune response as a result of inflammaging [48]. In contrast to previous research by Jylhävä et al. [9,10], our study did not find any significant difference in plasma cfDNA between young and elderly subjects. However, Jylhävä and colleagues recruited nonagenarians, aged from 90 to 99 years old, while our elderly participants were ~70 years old. In agreement with our results, Tosevska et al. [35] showed no change in plasma cfDNA levels in older adults following a 6-month resistance training, although the literature has recently demonstrated that both acute aerobics and resistance exercise elicit an elevation in the circulating cfDNA as a potential biomarker for obesity-associated inflammatory responses [49] and also muscle damage-related performance decrement [50], respectively. Since skeletal muscle is the primary source of cfDNA release to exercise, future research should investigate the effects of training modalities and intensities/durations to optimize the health benefits of physiological adaptation attributable to age-related muscle loss.

Senescent cells secrete bioactive exosomes (~30–150 nm) with the ability to induce paracrine senescence [51], pro-tumorigenic effects [52,53], or osteoarthritis [54]. Although we did not measure the plasma exosome particle concentration, the baseline level of total exosome proteins was equivalent between young and elderly groups. However, Eitan et al. [15] examined the impact of aging on the plasma EV (exosome and microvesicle) concentrations, demonstrating that elderly individuals have a lower level of plasma EVs than young individuals, possibly due to increased internalization by recipient cells (e.g., B cells and monocytes). While the mechanisms of these age-mediated EVs changes still remains to be elucidated, muscle-derived exosomes can use both paracrine and endocrine signaling to alter muscle homeostasis and communicate with other tissues, respectively [55]. Our systematic review has recently provided the scientific evidence regarding the effects of acute and chronic exercise on exosome biology [56]. In general, the literature has shown an increase in exosome release, regardless of exercise protocols, in different animal [24,26,29,31] and human [24,28,30] research. In the comparison of exercise protocols in human studies, the analyses of our systematic review showed inconclusive results in the release of plasma exosomes. For example, exosomes were released into circulation following a single bout of flywheel exercise [28], whereas no change in the exosome level was found following completion of 1-year rowing training [25]. With limited research evidence regarding the effect of chronic exercise, the absence of this modification in the exosome release could potentially be attributable to different exercise training protocols.

Regarding the measurements of exosome cargo, the present study demonstrated a lower expression of CD63 in elderly subjects than young subjects, with no difference in the levels of Flot-1, CD9, CD81, CD14, and VDAC1. Eitan and colleagues [15] have observed an elevated expression in CD63 and CD9, as well as monocyte-related antigen CD14, along with increased monocyte activation by isolated plasma EVs from older adults, suggesting an immunomodulatory role of EV-mediated inflammaging. Gomes de Andrade et al. [57] reported an increased CD63 level in EVs from the cerebrospinal fluid, whereas a decrease was found in the plasma level in aged rats. With limited literature examining the effects of exercise training in humans, our 8-week resistance training showed an attenuation of CD63 expression in trained subjects compared to the controls, whereas the expression of CD63 was increased in response to acute cycling exercise in young adults [58]. In animal studies, the majority of acute bouts of exercise [31] or short-term training (3-week swimming [24] and 2-week running [21]) elicited an increase in exosome markers (CD63, CD81, and Flot-1), regardless of the methods of isolation, characterization and phenotyping methodology, and exercise protocols (reviewed in [56]). The current literature has not provided sufficient evidence that healthy elderly subjects would have a large variation of CD63 response in such a short period of time. However, elevated levels of CD63 have been found in patients with cancers [59] and human immunodeficiency virus (HIV) infection [60]. An increase in CD63 on the surface of basophils cells has also been associated with allergic reactions or diseases [61]. Although all subjects remained healthy throughout the experiment, we cannot rule out that certain pathologies related to pro-inflammatory or carcinogenic processes were developing without having shown any symptoms and, therefore, without having been diagnosed. Considering that a reduction in the circulating exosomes could be due to both a lower release and/or a higher cell uptake, our 8-week resistance training protocol might be effective in improving the immune response in the prevention of age-related elevated CD63 response.

Finally, CD9 and CD81 are tetraspanins with similar functions as CD63 in immune cell recruitment and migration [62]. However, this study did not observe any changes with resistance training. Only one research has evaluated the effect of exercise on the release of CD9 in human plasma exosomes with an elevation observed following acute bouts of incremental cycling [58]. Chaturvedi et al. [29] reported an increase of CD81 in heart and serum-isolated exosomes in response to exercise training, along with the expression of Flot-1 protein after an 8-week treadmill running in diabetic mice, whereas no change of CD81 was shown following a 4-week swimming in rats [25]. Furthermore, our results showed no changes in exosome-carried CD14 and VDAC1 proteins with training. The exercise effect on the level of CD14 from human plasma-isolated exosomes was previously investigated, but different results were reported based on the use of the phenotyping technique [58]. Even though this study is the first to examine the effect of exercise on the level of exosome VDAC1 with no observation of any changes, future studies should discover the effective training interventions (aerobic or resistance) in the modulation of exosome markers (Flot-1, CD9, and CD81) and exosome-carried proteins (CD14 and VDAC1) to understand their exact roles associated with cardiometabolic conditions in the elderly.

## 5. Conclusions

This study showed a potential reduction in the inflammatory signaling through the attenuation of exosome CD63 expression in the elderly following an 8-week resistance training, without modification of plasma miR-146a-5p and cfDNA. However, this work has several limitations that are worth mentioning. For the evaluation of the exosome release to plasma, we presented only the exosome protein concentration. Since the release of exosome cargo can vary with exercise, further studies are needed to perform particle counting. Furthermore, the levels of miR-146a-5p and cfDNA in exosomes should be measured, which can expand the knowledge related to the expression of the selected exosome cargo. Finally, future research with an extended training protocol and the inclusion of exosome phenotyping are warranted to elucidate the roles of these inflammatory mediators on cellular aging and associated diseases.

## Figures and Tables

**Table 1:** Participant anthropometric characteristics.

	Young (*N* = 12)	CG (*N* = 10)	TG (*N* = 28)	*p*-Value ^#^
	Mean ± SEM	Mean ± SEM	Mean ± SEM	
**Age (years)**	22.3 ± 2.1	73.6 ± 0.8	72.6 ± 0.4	0.209
**Height (cm)**	176.4 ± 1.7	155.6 ± 3.2	163.3 ± 2.3	0.088
**Weight (kg)**	78.6 ± 2.4	69.8 ± 4,4	73.9 ± 3.1	0.516
**BMI (kg/m^2^)**	24.1 ± 1.5	28.9 ± 1.9	27.4 ± 0.8	0.406

BMI = body mass index; CG = control group; TG = trained group; ^#^ *p-*value CG vs. TG.

**Table 2:** Effect of an 8-week resistance training on muscular strength.

	CG (% Change)	TG (% Change)	*p*-Value
	Mean ± SEM	Mean ± SEM	
**1RM bench press seated (kg)**	−11.744 ± 4.415	32.672 ± 4.463	<0.001 *
**1RM leg extension (kg)**	14.343 ± 7.585	25.189 ± 3.021	0.040 *

CG = control group; TG = trained group; 1RM = one-repetition maximum; * *p* < 0.05.

**Table 3:** The baseline levels of miR-146, cfDNA, and total exosome protein content between young and elderly groups.

	Young (*N* = 12)	Elderly (*N* = 38)	*p-*Value
	Mean ± SEM	Mean ± SEM	
**miR-146a-5p (log10-scale)**	3.948 ± 0.482	4.3151 ± 0.214	0.618
**cfDNA (ng/mL)**	2277.079 ± 63.658	2443.279 ± 56.015	0.114
**Total exosome protein (µg/µL)**	5.022 ± 0.382	6.000 ± 0.328	0.140
**CD9 (O.D.)**	0.904 ± 0.179	1.049 ± 0.088	0.262
**CD14 (O.D.)**	1.850 ± 0.597	1.582 ± 0.164	0.486
**CD63 (O.D.)**	1.690 ± 0.218	1.499 ± 0.341	0.014 *
**CD81 (O.D.)**	1.311 ± 0.295	1.101 ± 0.089	0.856
**Flot-1 (O.D.)**	1.879 ± 0.399	1.633 ± 0.365	0.125
**VDAC1 (O.D.)**	0.958 ± 0.152	1.530 ± 0.184	0.078

CD = cluster of differentiation; cfDNA = cell-free DNA; O.D. = optical density; VDAC1 = voltage-dependent anion channel 1; * *p* < 0.05.

**Table 4:** The baseline levels of miR-146, cfDNA, total exosome protein content, and exosome cargo between CG and TG.

	CG (*N* = 10)	TG (*N* = 28)	*p* Value
	Mean ± SEM	Mean ± SEM	
**miR-146a (log10-scale)**	4.728 ± 0.224	4.150 ± 0.280	0.416
**cfDNA (ng/mL)**	2416.217 ± 156.234	2451.978 ± 56.499	0.376
**Total exosome protein (µg/µL)**	6.593 ± 0.587	5.789 ± 0.391	0.317
**CD9 (O.D.)**	1.053 ± 0.209	1.048 ± 0.095	0.777
**CD14 (O.D.)**	1.481 ± 0.230	1.623 ± 0.214	0.728
**CD63 (O.D.)**	1.397 ± 0.299	1.092 ± 0.097	0.584
**CD81 (O.D.)**	1.027 ± 0.156	1.128 ± 0.108	0.507
**Flot-1 (O.D.)**	2.207 ± 0.884	1.420 ± 0.383	0.608
**VDAC1 (O.D.)**	1.151 ± 0.268	1.685 ± 0.230	0.151

CD = cluster of differentiation; CG = control group; O.D. = optical density; TG = trained group; VDAC1 = voltage-dependent anion channel 1.

**Table 5:** Effects of an 8-week resistance training on plasma miR-146, cfDNA, total exosome protein content, and exosome cargo between CG and TG.

	CG (% Change)	TG (% Change)	*p*-Value
	Mean ± SEM	Mean ± SEM	
**miR-146a-5p (log10-scale)**	−2.681 ± 9.184	13.945 ± 8.659	0.360
**cfDNA (ng/mL)**	−1.370 ± 1.431	−3.057 ± 1.159	0.396
**Total exosome protein (µg/µL)**	−6.663 ± 8.173	−3.990 ± 8.173	0.947
**CD9 (O.D.)**	26.147 ± 15.547	17.668 ± 9.393	0.571
**CD14 (O.D.)**	−2.586 ± 13.333	103.368 ± 71.834	0.192
**CD63 (O.D.)**	42.541 ± 14.044	6.786 ± 5.524	0.027 *
**CD81 (O.D.)**	4.681 ± 7.292	5.985 ± 6.481	0.842
**Flot-1 (O.D.)**	49.712 ± 31.691	31.018 ± 12.679	0.784
**VDAC1 (O.D.)**	0.551 ± 11.800	34.428 ± 18.493	0.433

CD = cluster of differentiation; CG = control group; O.D. = optical density; TG = trained group; VDAC1 = voltage-dependent anion channel 1; * *p* < 0.05.

## Data Availability

The data presented in this study are available on request from the corresponding author.
